# Well-Being of Ambulatory Adults With Cerebral Palsy: Education, Employment, and Physical Function of a Cohort Who Received Specialized Pediatric Care

**DOI:** 10.3389/fneur.2021.732906

**Published:** 2021-09-20

**Authors:** M. Wade Shrader, Chris Church, Nancy Lennon, Thomas Shields, Jose J. Salazar-Torres, Jason J. Howard, Freeman Miller

**Affiliations:** Department of Orthopedics, Nemours duPont Hospital for Children, Wilmington, DE, United States

**Keywords:** cerebral palsy, adults, health, education, employment, independence

## Abstract

**Introduction:** The transition from pediatric health care and school systems presents enormous challenges for young adults with cerebral palsy (CP). The lack of strong societal support during this seminal life event is well-documented and leads many adults with CP to struggle with independence, higher education, and employment. Despite the relatively high prevalence of CP, information about the experiences and function of adults with CP in our society continues to be limited. The purpose of this project was to describe well-being by assessing education, employment, physical function, walking activity, and utilization of health care in an ambulatory adult cohort with CP who received specialized pediatric care at our center.

**Method:** In this Institutional Review Board-approved prospective study, we invited former patients from our tertiary care pediatric CP center to complete a set of patient-reported outcomes including (1) the Patient-Reported Outcomes Measurement Information System domains of physical function and pain interference, (2) the Satisfaction with Life Scale, and a project-specific demographic questionnaire about education, employment, income, independence, pain, and health care utilization. Participants also wore a pedometer for 8 days to monitor community walking activity. Chi-squared pairwise or *t*-tests were used as appropriate to compare survey responses and walking activity data between three groups: participants who self-reported, those who reported by proxy, and published normative data from age-matched typically developing adult (TDA) samples.

**Results:** One hundred twenty-six adults with CP consented to participate; 85 self-reported [age 29.7 ± 4.3 years; Gross Motor Function Classification System: I (28%), II (47%), and III, (25%)] and 41 reported by proxy [age 29.7 ± 4.1 years; Gross Motor Function Classification System: I (10%), II (68%), and III (22%)]. For the group who self-reported, high school graduation rate (99%) was similar to TDA (92%; *p* = 0.0173) but bachelor's degree achievement rate (55%) was higher than TDA (37%; *p* < 0.001). Despite more advanced education, the unemployment rate in this group was higher than national levels at 33% and was associated with high utilization of Social Security Disability Insurance (33%). Within the self-reporting group, 13% required a caregiver. For the group who reported by proxy, educational levels (73% high school graduates, 0 bachelor's degree) were lower than the general population (*p* < 0.001) and unemployment was higher than the national level, at 64%. Unemployment in this group was associated with high utilization of Social Security Disability Insurance (85%). Within the proxy-reporting group, 71% required a caregiver. The full cohort demonstrated lower levels of physical function according to the Patient-Reported Outcomes Measurement Information System and less community walking activity compared with TDA references (*p* < 0.001). This cohort of adults with CP reported significantly higher frequency of chronic pain (48 vs. 12% for TDA; *p* < 0.001), but less pain interference with daily activities than TDA based on Patient-Reported Outcomes Measurement Information System results (*p* < 0.001). This cohort reported good to excellent overall health (93%) and high utilization of primary care (98%), but limited utilization of specialty care, specifically orthopedic care (21%) and physical therapy (15%).

**Discussion:** This cohort of adults with CP had similar levels of education as the general population, but had relatively high rates of unemployment, caretaker need, and Social Security Disability Insurance utilization. Although chronic pain was frequent, the impact of pain on work and independent living did not exceed reports from a typically developing reference. Better targeted societal resources for adults with physical disabilities are urgently needed to allow equitable access to employment, promote opportunities for independence, and enable full participation in community life.

## Introduction

Cerebral palsy (CP) describes a group of permanent disorders of the development of movement and posture, which cause activity limitation and are attributed to non-progressive disturbances that occurred in the developing fetal or infant brain. The motor disorders of CP are often accompanied by disturbances of sensation, perception, cognition, communication, and behavior; by epilepsy; and by secondary musculoskeletal problems (1). Cerebral palsy is known to cause motor dysfunction leading to impairments that persist throughout the lifespan (2). These impairments can have an impact on mobility and a variety of factors that influence overall quality of life and well-being. With appropriate medical and supportive care, life expectancies for ambulatory adults with CP can approach that of typically developing adults (TDA). Hence, understanding trajectories of well-being and function for adults with CP is important to determine barriers that prevent individuals from achieving their maximum societal potential.

Currently, limited research is available on self-reported outcomes of adults with CP, whether functional in nature or related to quality of life (2, 3). From a functional standpoint, orthopedic surgery is widely applied during childhood to help improve long-term mobility and participation. These treatments, however, may only prevent or retard the development of further functional deterioration and relative gains that may not affect a patient's independence, education goals, or levels of employment as adults (4). As such, more research throughout the lifespan of individuals with CP is needed to identify barriers and develop solutions that serve to improve long-term outcomes–both functional and societal–for adults with CP.

Conflicting evidence exists related to achievements in education and employment for adults with CP (5–11). Reports of university degree completion varied widely at 2% (6), 5% (7), and 24% (8). A study from South Africa found that 50% of adults with CP had enrolled in post-secondary education, but the main source of income for 37% of the participants was governmental disability benefits (9). Employment rates for adults with CP also vary, reported at 79% (10), 68% (5), 45% (11), and 36% (6).

Access to primary and specialized health care is important for well-being. There is conflicting evidence in the literature regarding adequate access to health care for adults with CP in the United States. For instance, a study by Gannotti et al. (4) found that over half of adults with CP reported having “easy” to “very easy” access to health care with only a small number classifying their access to health care as being difficult. Orlin et al. identified several barriers for adults with CP to access specialized health care, including relying on family or caregivers for transport, communication difficulties, and issues relating to follow-up (12).

A few studies have investigated quality of life (QoL) in adults with CP (13–16). Similar to studies focused on functional mobility, findings relating to life satisfaction, depression, and well-being have been inconsistent. One study suggested that adults with CP maintain childhood levels of mobility function in young adulthood, are satisfied with social roles, and have minimal pain (3). In their population-based study, Morgan et al. reported that health status and well-being in adults with CP is below average compared with TDA (14). By contrast, Jarl et al. reported high health-related QoL in adults with CP overall, with reduced QoL only for those with severe motor dysfunction and pain (13). Others reported that limited functional mobility is not associated with mental health impairment (3, 15, 16). Pain, which has been linked to decreased QoL, is highly prevalent in CP (30–80% of adults) (17–19).

The purpose of this project was to assess independence, education, employment, walking activity, pain, utilization of health care, and physical function in an adult cohort of former patients from a pediatric CP specialty center.

## Materials and Methods

Potential study participants were identified from a historical database from the authors' institution. Inclusion criteria were individuals who (a) were between the ages of 25 and 45 years; (b) had a diagnosis of CP; and (c) were functioning at Gross Motor Function Classification System (GMFCS) level I, II, or III. The study was approved by our institutional review board (# 1115672).

### Patient-Reported Outcomes

Participants were asked to complete, with or without caregiver assistance, an online demographic survey and two domains of the Patient-Reported Outcomes Measurement Information System (PROMIS) tool, including (1) physical function v2.0 and (2) pain interference v1.1. The PROMIS is a rigorously constructed, generalizable, and clinically relevant set of patient-reported outcomes developed by the National Institutes of Health (20). Additionally, the survey consisted of questions that collected demographic data (such as sex, age, ethnicity, race, etc.), highest level of education, employment, personal and household income levels, frequency of pain, health care provider utilization, and life satisfaction (21, 22).

### Walking Activity

To monitor community-based walking activity, participants were given a Food and Drug Administration approved class-2 activity monitoring device (Modus StepWatch, Edmonds, WA USA), to track the participant's walking activity for 8 consecutive days. The device was calibrated to each adult's specific gait pattern in our gait analysis laboratory following the guidelines of the manufacturer to verify accuracy (23).

### Normative Comparison

Whenever possible, we compared our data with published results for age-matched TDA in the United States. Education was compared with data collected through the United States Census in 2017 (24) for adults aged 25–34 years. Information on employment and the rate of Social Security Disability Insurance was compared with the United States Bureau of Labor Statistics report (25). Income levels for TDA from 2017 were obtained from the Income and Poverty in the United States: 2019 report (26). An age-matched (30–39 years old) cohort of 193 TDA living in the United States was used for comparison with the average strides per day collected from adults with CP (27). In 2012, pain was self-reported in a study population consisting of over 8,000 adults in the United States, where 11.2% of the adults reported chronic pain occurring on a daily basis (28). Normative values for PROMIS pain interference and physical function were obtained from a published data bank source of 1,700–15,903 TDA adults (20). Satisfaction with Life Scale results were compared with published TDA data (22).

### Statistics

Pairwise comparisons, chi square, or *t*-tests were used as appropriate to compare responses between self-reporting adults (*n* = 85), proxy-reporting adults (*n* = 41), and normative data for TDA. Statistical significance was set at *p* < 0.0167 to account for multiple (three-way comparisons). All statistical analyses were performed using R-Studio version 1.4.1106 (29).

## Results

**Figure 1:**
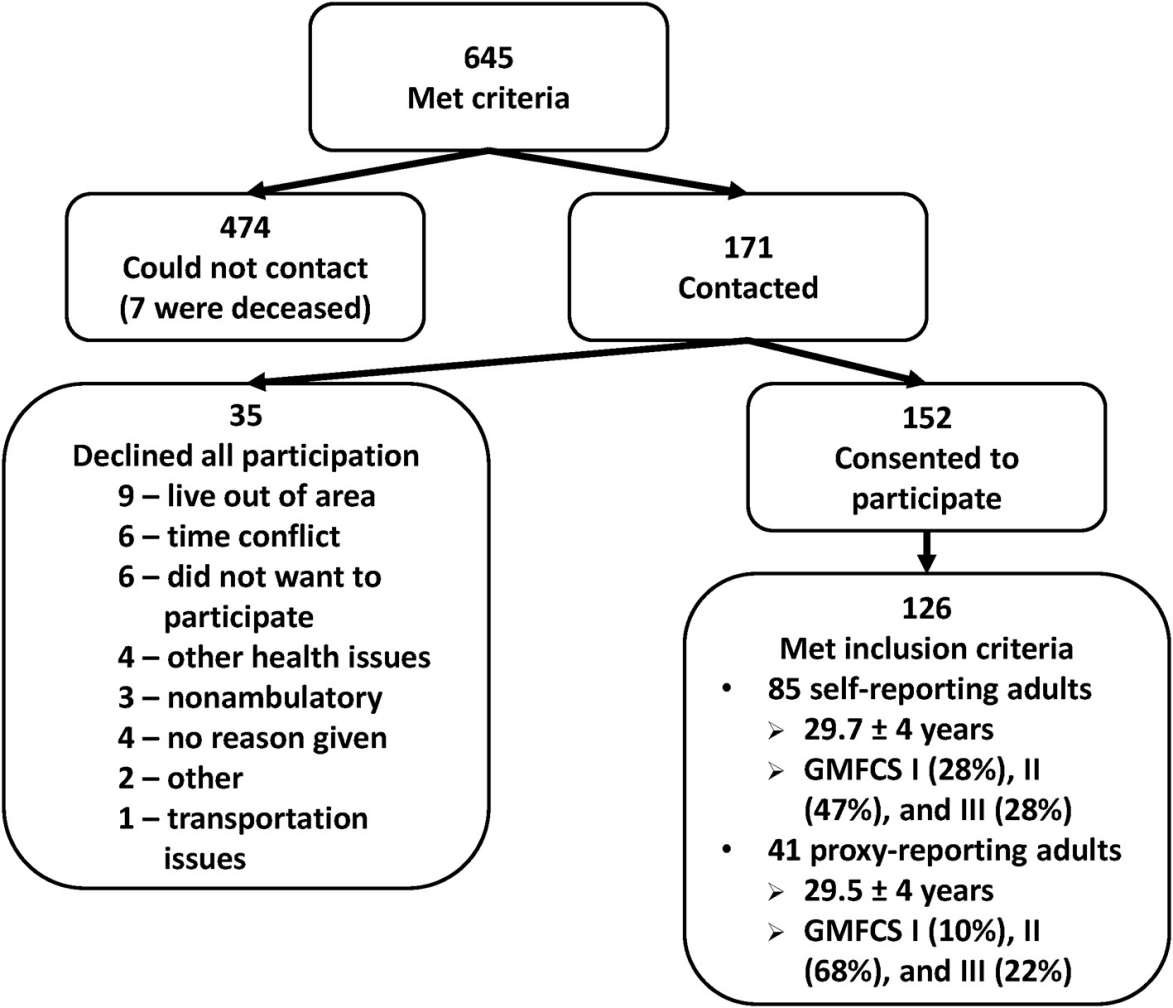
Breakdown of 645 adults who met the inclusion criteria and were invited to participate in the study. GMFCS, Gross Motor Function Classification System.

A total of 645 adults who met the inclusion criteria were identified from historical databases and invited to participate in the study. Of these, we successfully contacted 171, and a total of 152 adults with CP agreed to participate in the study (see Figure 1). Twenty-six respondents were excluded due to having incomplete survey responses or not functioning at GMFCS level I, II, or III. Participants were placed in two groups, those who completed the survey/questionnaire by themselves (self-reported) and those who used an assistant to answer the questions (proxy-reported). A total of 85 participants were included in the self-reported (SR) group (41 female, 29.7 ± 4.3 years old). This group self-identified ethnically as 88% White, 9% Black or African American, 1% Japanese, and 1% as “Other.” The GMFCS distribution was I (28%), II (47%), and III (25%), and was not significantly different compared with the 645 eligible participants (*p* > 0.1).

A total of 41 participants were included in the proxy-reported (PR) group [15 females (37%), 29.7 ± 4.1 years old]. This group self-identified ethnically as 73% White, 20% Black or African American, 2% Korean, and 5% as “Other.” The GMFCS distribution was I (10%), II (68%), and III (22%).

### Socioeconomic Factors

Participants in the SR group were found to have similar or higher levels of education compared with the TDA population, in contrast with the PR group who were found to have lower levels of academic achievement (see Table 1). Eighty-four participants in the SR group (99 vs. 92% of TDA; *p* = 0.0173) graduated from high school and 48 (57 vs. 37% of TDA; *p* < 0.001) obtained a bachelor's degree or higher. Only 30 participants in the PR group (73%) were found to have graduated from high school and none had attained a level of higher education (bachelor's degree or higher; *p* < 0.001 compared with the SR group and TDA).

**Table 1:** Socioeconomic factors: education, employment, and income of adults with cerebral palsy compared with non-disabled adult populations.

**Outcome**	**Self-report** **(*n* = 85)**	**Proxy-report** **(*n* =41)**	**TDA**	**SR vs. PR** ***p*-value**	**SR vs. TDA** ***p*-value**	**PR vs. TDA** ***p*-value**
Graduated high school, %	99	73	92	<0.001	0.0173	<0.001
Attained bachelor's degree or higher, %	57	0	37	<0.001	<0.001	<0.001
Employment, %	68	37	96	<0.001	<0.001	<0.001
Full-time/part-time, %	68/32	27/73	80**/**20	<0.001	0.003	<0.001
Personal income [Mean (SD)]	$36,743 ($32,308)	$14,193 ($23,169)	$35,001	<0.001	–	–
Household income [Mean (SD)]	$86,394 ($58,185)	$73,451 ($60,876)	$86,992 ($892)	0.259	0.925	0.154
Utilization of SSDI, %	32	85	–	<0.001	–	–

–* indicates not applicable. TDA, typically developing adults; PR, proxy-report; SD, standard deviation; SR, self-report; SSDI, Social Security Disability Insurance*.

Similarly, participants in the SR group were found to have a comparable level of personal and household income as TDA, while participants in the PR group had significantly reduced personal income compared with the SR group (*p* < 0.001). In terms of unemployment rate, both groups in this study showed higher levels of unemployment compared with the 4.4 ± 0.2% unemployment rate reported for adults in the United States through 2017 (*p* < 0.001) (25). Additionally, adults with CP in the PR group showed higher levels of Social Security Disability Insurance use than the SR group (*p* < 0.001).

### Independence and Physical Capacity

Regarding levels of functional independence, the PR group showed a greater need for a caregiver than the SR group (*p* < 0.001) (see Table 2). Adults with CP showed significantly higher levels of chronic pain, 47.1% (SR) and 53.7% (PR), compared with 11.2% found in TDA (*p* < 0.001), but demonstrated less pain interference in daily activities than TDA reported according to PROMIS results (*p* < 0.001).

**Table 2:** Independence and physical capacity.

**Outcome**	**Self-report** **(*n* = 85)**	**Proxy-report** **(*n* = 41)**	**TDA**	**SR vs. PR** ***p*-value**	**SR vs. TDA** ***p*-value**	**PR vs. TDA** ***p*-value**
Need a caregiver, %	13	71	–	<0.001	–	–
Spasticity, %	66	66	–	1.00	–	–
Joint/muscle pain, %	72	61	–	0.214	–	–
Chronic pain, %	47	54	11	0.487	<0.001	<0.001
SWLS [Mean (SD)]	26.2 (7.0)	22.5 (7.5)	20–24 (Range)	0.009	–	–
PROMIS pain interference [Mean (SD)]	50.7 (9.3)	49.2 (8.1)	55.9 (10.8)	0.356	<0.001	<0.001
PROMIS physical function [Mean (SD)]	44.5 (9.7)	35.5 (8.9)	50 (10)	<0.001	<0.001	<0.001
Walking activity [strides/day; Mean (SD)]	2,679 (1,992)	1,727 (269.7)	5,127 (2,834)	<0.001	<0.001	<0.001

–* indicates not applicable. TDA, typically developing adults; PR, proxy-report; PROMIS, Patient-Reported Outcomes Measurement Information System; SD, standard deviation; SR, self-report; SWLS, Satisfaction with Life Scale*.

In terms of walking activity, 75 individuals from the SR group and 39 from the PR group had valid data from the StepWatch device. Walking activity was significantly lower in both groups compared with a published cohort of 193 TDA in the United States (*p*-value < 0.001) (27). These results were in keeping with lower scores on the PROMIS physical function domain observed in both groups compared with TDA (*p* < 0.001).

### Primary and Specialty Care Utilization

There were no significant differences in primary and specialty care use between groups of people with CP (*p* > 0.1) (see Table 3). Our results indicate that adults with CP had relatively low utilization of orthopedic specialists (~20% in both groups) or physical therapists (~15% in both groups). Women's responses indicated that slightly over half visited an obstetrician-gynecologist. Nevertheless, around 90% of both groups classified their overall health as “good to excellent.”

**Table 3:** Primary and specialty care utilization by adults with cerebral palsy.

**Outcome**	**Self-report (*n* = 85)**	**Proxy-report (*n* = 41)**	***P*-values**
Visit an orthopedic specialist, %	20.0	19.5	0.947
Visit a physical therapist, %	15.3	14.6	0.918
Visit a primary care physician, %	97.6	100	0.317
Visit a dentist, %	75.3	87.8	0.104
Visit an obstetrician-gynecologist (females), %	67.3	53.3	0.335
Good to excellent health, %	92.9	87.8	0.343

## Discussion

The transition from adolescence to adulthood is a seminal life event. For youth with CP, the loss of pediatric health care and school-based support systems presents enormous challenges. A lack of strong societal support mechanisms is well-documented for people with disabilities and leads many young adults with CP to struggle with independence, higher education, and employment (2, 5–11, 30). These observations have been documented in other countries, such as Norway where individuals with CP had lower odds of completing upper secondary education and had higher odds of receiving a disability pension (31). Similarly, a study from Latvia showed only 9% of adults having a paid job whereas 44% were still financially dependent as adults (32). This is consistent with several other studies reporting low employment rates in adults with CP (5, 6, 10, 11). Despite the relatively high prevalence of CP, information on the life experiences and function of adults with this condition in our society is limited. Conflicting and limited evidence is available related to mobility, quality of life, and the factors that influence these issues in adults with CP (3, 13–16). Such knowledge could inform both comprehensive transition programs and better targeted societal mechanisms to support adults with childhood onset conditions such as CP.

From a socioeconomic perspective, there were clear differences in this cohort between those who self-reported and those who did not. Educational achievement and employment rates were higher in the SR group compared with the PR group. In terms of work “quantity,” full-time employment was more prevalent than part-time in the SR group, whereas part-time employment was more commonly reported in the PR group.

When compared with the TDA reference, both the SR and PR groups had significantly lower employment rates. While adults with CP in the SR group had similar high school and higher post-secondary education attainment rates as TDA, there was a significantly lower employment rate than in the TDA reference. This suggests the presence of societal barriers to employment opportunities for adults with CP.

Our results on educational attainment compared with the TDA population are in contrast with other studies where lower levels of advanced educational attainment were found (7, 9). Some of the variability in education and employment reports could be related to the frequency of cognitive involvement in each study sample. Many of the differences between the SR and PR groups are thought to be related to comorbid cognitive impairments that we did not fully capture in the demographic survey.

It has been reported that adults with CP face declining health with aging related to increased levels of pain (2, 30, 33, 34). For instance, a study on self-reported health outcomes found that 63% of adults with CP report chronic pain (35). This is consistent with the current study findings where, compared with a cohort of TDA, both groups of adults with CP (SR and PR) demonstrated higher frequency of chronic pain, with lower levels of physical function and community-based walking activity. Despite the high incidence of chronic pain, this cohort of adults with CP reported less pain interference with daily activities compared with TDA and had average to above average satisfaction with life. Similar findings on pain interference levels were reported by Flanigan et al. for a group of adults with CP (36).

Utilization of specialized musculoskeletal care by the young adults with CP in this study was much lower compared with the highly specialized pediatric services provided during childhood, specifically, orthopedics, and physical therapy. While we could not confirm that low utilization was related to lack of access, participants frequently reported that they could not find adult providers specializing in CP care.

It has been hypothesized in the literature that access to specialty health care for adults with CP is limited because CP is considered to be a static condition with a focus on care during childhood (2, 30, 37). There is conflicting information in the literature about the accessibility to specialized health care for adults with CP. Roquet et al. found that adults with CP have low utilization of rehabilitative physical health care (37), whereas Gannotti et al. found that most adults with CP had easy access to general health care (4).

Children with CP have higher utilization of specialty care, the focus of which is optimizing mobility function and musculoskeletal health, compared with typically developing youth. The lack of quality rehabilitation and orthopedic care for adults with CP could hinder the maintenance of functional mobility since orthopedic sequelae persist throughout the lifespan and continue to impact motor performance. Compounding this on the medical side, a high prevalence of comorbidities in persons with CP is associated with decreased overall health in adulthood (2, 30, 33, 34).

### Limitations

This study was not population-based and included ambulatory adults with CP who were previously treated at the authors' institution. Therefore, our results should be interpreted with caution when extrapolating to adults with CP in other locales. GMFCS scores were given at the adult visit, but the classification has not been validated in that population. Another limitation in this study was the inability to distinguish between participants in the PR group who had a substantial cognitive or fine motor skill impairment as the reason for a proxy requirement and those who did not. Future research into the health-related outcomes and community participation of adults with CP should focus on identifying and removing barriers as well as developing effective care and support mechanisms throughout the lifespan for people with CP.

## Conclusion

The cohort of young adults with CP in this study had similar levels of higher education as the general population and had good access to primary health care. Despite this, there were relatively high rates of unemployment, caretaker need, and SSDI utilization. Although reports of pain were frequent, the pain did not interfere with physical activities and did not limit satisfaction with life compared with an age-matched reference. Improvements in societal and medical resources for adults with childhood onset physical disabilities such as CP are urgently needed to allow equitable access to employment and independent living opportunities and promote full societal integration.

## Data Availability Statement

The raw data supporting the conclusions of this article will be made available by the authors, without undue reservation.

## Ethics Statement

The studies involving human participants were reviewed and approved by Nemours Institutional Review Board. The patients/participants provided their written informed consent to participate in this study.

## Author Contributions

MS, CC, NL, JH, and FM contributed to the conception and design of the study. MS, CC, NL, TS, JS-T, JH, and FM contributed to the methodology of the study. JS-T and JH contributed to software design and procurement for the study. CC, NL, TS, JS-T, and JH participated in the validation of the data. MS, CC, NL, TS, JS-T, and FM participated in the formal analysis of the data. MS, CC, TS, JS-T, and FM participated in investigation of the data and provided resources for the study. MS, CC, TS, JS-T, and JH curated the data for the study. MS and TS wrote the original draft of the manuscript. MS, CC, NL, and FM provided visualization for the study and acquired the funding for the study. MS, CC, and FM provided supervision for the study. CC provided project administration for the study. All authors contributed to manuscript revision, read, and approved the submitted version.

## Funding

This study was funded by the Pedal with Pete Foundation.

## Conflict of Interest

The authors declare that the research was conducted in the absence of any commercial or financial relationships that could be construed as a potential conflict of interest. The reviewer HG declared a past collaboration with two of the authors MS and FM to handling Editor.

## Publisher's Note

All claims expressed in this article are solely those of the authors and do not necessarily represent those of their affiliated organizations, or those of the publisher, the editors and the reviewers. Any product that may be evaluated in this article, or claim that may be made by its manufacturer, is not guaranteed or endorsed by the publisher.

## Abbreviations

CP: cerebral palsy
GMFCS: Gross Motor Function Classification Scale
TDA: non-disabled adults
PR: proxy-reported
PROMIS: Patient-Reported Outcomes Measurement Information System
SR: self-reported.
